# Fetal–Fetal and Fetal–Maternal Microchimerism: Insights from Mammalian Placental Biology

**DOI:** 10.3390/jdb14020019

**Published:** 2026-04-28

**Authors:** Jorge A. De los Santos Funes

**Affiliations:** Department of Agricultural Sciences, Texas State University, San Marcos, TX 78666, USA; delossantos@txstate.edu

**Keywords:** trophoblast, multinucleated cells, cell-free fetal DNA, maternal plasma, placenta, pregnancy, fetal, microchimerism

## Abstract

Feto-maternal microchimerism (Mc) refers to the exchange of cells between the fetus and mother, and fetal–fetal Mc to the exchange between fetuses during pregnancy. This phenomenon occurs across mammalian species, including humans, mice, and cattle. Key data on Mc cells and theoretical considerations regarding the presence of fetal-derived material, such as trophoblast cells, cell-free fetal DNA (cffDNA), and exosomes in maternal blood are summarized. This review aims to first, synthesize current knowledge on feto-maternal and fetal–fetal Mc across mammals, second, address three core questions: how and where Mc has been demonstrated in animals, what techniques have been used over time to detect fetal-derived material and Mc, and how placental structures influence the frequency of Mc. Finally, it aims to identify gaps in the literature for species such as horses, goats, and pigs. This article concludes that Mc is a widespread phenomenon among mammals, but detection methods and reported frequencies vary significantly by species and placental type. A biological model is presented in this article in which multinucleated trophoblast cells undergo apoptosis, releasing cffDNA that enters the maternal blood circulation after multinucleated trophoblast invasion. Advances in molecular biology technology have improved the ability to detect fetal-derived material, cells, DNA, and exosomes in maternal blood. However, notable research gaps remain for Mc in horses, goats, and pigs, highlighting the need for targeted studies to better understand species-specific patterns or a general biological model.

## 1. Introduction

Microchimerism, or Mc, refers to the biological phenomenon wherein individuals have a distinct cell population of a different origin. Mc is well described in blood transfusion and organ transplantation in medicine [1]. Particularly, the term feto-maternal Mc in this review refers to the exchange of cells or nucleic acids derived from the placenta to the maternal bloodstream.

Feto-maternal Mc has been found to occur naturally across all eutherian (placental mammal) species. This article also covers fetal-to-fetal Mc. This has been reported to take place through the placenta and has significant theoretical and practical relevance [2]. This article builds on that foundation by incorporating new insights from molecular and cell biology, providing an updated understanding based on 70 years of research. Phylogenetic analysis shows that the invasiveness of the placenta cells in mammals is strongly associated with four clades: Afrotheria, Xenarthra, Euarchontoglires, and Laurasiatheria, represented by modern elephants, armadillos, monkeys, and cows, respectively. This phylogenetic relationship can be linked with the plate tectonics separation more than 100 million years ago [3]. Remarkably, this phylogenetic relationship is supported by the placenta invasiveness and structure [4,5], as well as molecular DNA sequencing data [6].

Trophoblasts are the specialized cells that surround the early embryo and diverge early from the pluripotent inner cell mass, unique to mammals [7]. These cells are presumed to be the primary contributors to microchimerism (Mc). The aim of this work is to identify gaps in the existing literature and to discuss a conceptual model that may explain the occurrence of Mc beyond the influence of the placental type of domestic animals.

## 2. Hemochorial Placentas

Several studies have associated feto-derived cells with positive and negative effects on maternal health. According to a review article, the positive and negative interactions can explain the contradictory scientific data from the literature [8]. For instance, placental cells help to heal after a cesarean delivery by releasing local Collagen I and III, and TGF-β3, molecules that promote local cell proliferation [9]. However, the molecular and cell migration signaling behind this behavior remains unclear. In vitro data prove that cell-free fetal DNA (cffDNA) can function as a damage-associated molecular pattern and trigger an inflammatory cytokine response that facilitates key processes associated with parturition [4]. In vivo data from humans have proven that cffDNA is detected in the maternal blood of a woman who has lost the fetus during pregnancy [10].

The evident maternal immunological response occurs during the second pregnancy when the first fetus provokes immunization against Rh antigens on the surface of the erythrocytes. It is well described in the scientific literature that antibodies from the mother can go across the placenta and enter the fetal circulation, with fatal consequences for the second fetus [11].

Feto-maternal Mc plays a crucial role in the maternal immune system. It has been proposed that maternal B cells and natural killer (NK) cells are associated with feto-maternal Mc, impacting the mother’s immunological cells, for instance, having a protective action against breast cancer [12]. The author explains this observation by the bidirectional transfer and persistence of Mc cells between mother and offspring, which may have lasting impacts on future pregnancies [13]. In contrast to the tissue repair hypothesis, other researchers have proposed that the presence of fetal cells after delivery can promote tumorigenic factors [14].

In 1893, Georg Schmorl, a German researcher, was the first person to find and describe multinucleated cells from the placenta in maternal blood and organs. He studied 17 women who died of hypertensive disorders during pregnancy [15]. Schmorl isolated and studied by microscope the morphological patterns of the cell mass found in organs and blood coagulation. He first described giant cells in the lungs. Comparing the morphology of the cells found in maternal blood and those within the placenta, he concluded that those under the microscope corresponded to the giant cells derived from the placenta [15]. Schmorl was one of the first researchers to ask about the biological meaning of placental cells in maternal blood and if they could survive in the maternal body.

The first report of fetal–maternal Mc has since been confirmed by various researchers across different laboratories, using diverse techniques and conducted at separate times. In the 1980s, Mueller and colleagues provided significant insights by isolating syncytiotrophoblast cells from the human placenta. They developed a monoclonal antibody, FDO46B (IgG1, κ), targeting an antigen present on the trophoblast membrane during the first trimester [16].

Building on this work, another study in Germany analyzed trophoblast cells in the maternal bloodstream of eleven pregnant women. Using magnetic cell sorting, the researchers isolated trophoblasts and compared their genetic profiles, including the HLA-DQ-A1 locus and D1S80 marker, with paternal genetic data to confirm fetal origin [17]. Together, these studies underscore the diverse approaches employed to validate the presence of fetal cells in maternal circulation.

Nevertheless, the researchers use the monoclonal antibody FDO46B to isolate fetal trophoblast cells from peripheral blood samples in 13 pregnant women at 34 weeks of gestation [18]. After isolating the cell by immunoprecipitation, they compared the amplification of Y chromosome markers by PCR and the sexing results of karyotyping techniques from chorionic villus samples [18].

Bianchi, working at the Boston Children’s Hospital (USA), used cytometric techniques and labeled the cells with a monoclonal antibody against the transmembrane glycoproteins transferrin receptors (TfR), which are typically expressed on undeveloped erythroid and rapidly dividing cells that include placenta-derived cells [19,20]. By molecular biology techniques, the isolated population was used to confirm the presence of Y chromosome DNA markers in humans. With this approach, Bianchi’s laboratory was able to isolate fetal nucleated erythrocyte cells from two women at 11 and 12 weeks of gestation [19,21].

In 1994, an Australian group collected fourteen peripheral blood samples of pregnant women between 9 and 18 weeks of gestation, using immunomagnetic beads coated with monoclonal antibodies. They isolated, in twelve out of fourteen samples, big cells and compared the morphologies with the placenta syncytiotrophoblast. They concluded that the cells isolated from maternal blood and placenta are morphologically the same under the microscope [22].

In 1997, a group of researchers from Hong Kong showed that 6.2% of total cffDNA in the maternal plasma, in humans, came from the male fetus using quantitative techniques such as real-time PCR [23]. Male fetal DNA sequences are present in the mother’s serum during the pregnancy and disappear rapidly postpartum, according to data from rhesus monkeys [24]. It is not surprising that stable fetal RNA is also present in maternal plasma [25]. It has been described that microRNAs, such as miR-515-3p, hsa-miR-517a, hsa-miR-517c, hsa-miR-518b, and hsa-miR-526b, are present in the last stage of gestation in maternal plasma. These microRNAs increase in concentration across the pregnancy, but the biological functions remain unclear [26]. In healthy women during pregnancy, some estimations suggest the presence of one placenta-derived cell per ml of maternal blood on average [27]. The blood samples were obtained from healthy pregnant women around 11 weeks of gestation. The cells were isolated by filtration and characterized by molecular biological techniques, such as short tandem repeats (STRs) and Y-specific chromosome markers, amplified by PCR [27]. Interestingly, the trophoblast migration and cancer metastatic processes share some characteristics. The signaling interaction between embryo-derived and endometrial cells resembles cancer metastatic tissue invasion [28,29]. Moreover, Paterlini-Bréchot developed a filtration technique for isolating placenta-derived cells in the mother’s blood, and then the technique was used to separate cancer cells in the blood [27,30].

Blood sampling allows the development of non-invasive techniques that have become popular in comparison to other technical procedures, such as chorionic villus sampling or amniocentesis. These procedures are invasive for both the mother and fetus and can potentially increase the risks of miscarriage [31,32]. This area of intense study is known in medical applications as a noninvasive prenatal diagnosis. Furthermore, some researchers predict that these technologies can open new opportunities to treat genetic disorders in the uterus [33].

In 2002, a study of 160 women—all of whom had at least one son and no history of autoimmune disease, miscarriages, or blood transfusions—reported that free fetal DNA can be present in maternal circulation 40 years after delivery of a male. Pregnant women expecting twins were excluded from the cohort [34]. In contrast to this, a Japanese laboratory using 10 samples from singleton pregnant women demonstrated that the number of fetal nucleated cells in maternal blood decreased after delivery [35].

The hemochorial model indicates that extracellular vesicle particles that carry free fetal DNA are cleared in the lungs [36]. Data from maternal rhesus monkeys, Macaca mulatta, proves as well in humans that the hemochorial placenta sheds off cells to the mother’s bloodstream [37]. Like humans, the cffDNA in rhesus monkeys also increases in plasma with the gestational stage [24]. In addition, a CD34 presumably from the fetus was reported as Mc in pregnant Macaca mulatta blood [38]. This finding demonstrates that the hemochorial type of placenta has similar dynamics in humans and non-human monkeys.

In conclusion, feto-maternal Mc plays a vital role in influencing and shaping the maternal immune system in the epitheliochorial human placenta. In addition, one recent review article defines “communicatome” as that which includes soluble factors, immune cells, and extracellular vesicle (EV) particles that travel from the placenta to the maternal blood [39].

## 3. What Is Known in Mice?

In mice, fetal cells have also been described in the brain, crossing the placenta and brain barriers. These cells can reach the brain of the mother and presumably have a neurological importance in mice [40]. Dawe et al. argue that this phenomenon can be a simple consequence of pregnancy without a biological explanation [41]. Three hypotheses can explain feto-maternal Mc in mice. First, trophoblast cells can be deported when they are close to maternal vessels and the intervillous space. Second, micro-traumatic hemorrhage can leak cells in both directions. Third, cell adhesion and transmigration across the placental barrier should contain specific receptors at the cell membrane [41].

**Figure 1:**
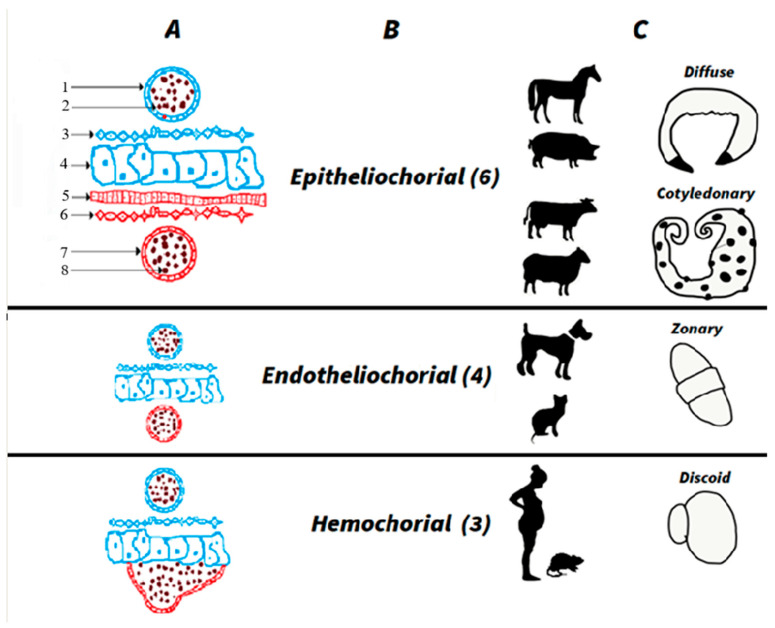
Diagram illustrating maternal blood vessels and placental structure. The illustration depicts the invasiveness of the placenta into the maternal endometrium, classified into three types: epitheliochorial, endotheliochorial, and hemochorial. Numbers in parentheses indicate the number of tissue layers separating the maternal bloodstream from the fetal placenta. (**A**) Tissue and cell types are represented in red (maternal tissues) and blue (placenta-derived cells). (**B**) Names of types with numbers indicating the number of tissue layers separating maternal and fetal bloodstreams. (**C**) Examples of animals corresponding to each placental type, with details on fully developed placental morphology. References: 1—endothelial cells (placental side), 2—red blood cells (placental side), 3—mesenchymal cells, 4—trophoblast cells, 5—epithelial cells (maternal side), 6—stroma cells, 7—endothelial cells (maternal side), 8—red blood cells (maternal side).

The fact that different cells can show up in maternal circulation besides the trophoblast, for instance, lymphocytes [42,43,44], erythrocytes [45,46], and the hematopoietic progenitor cells [47], goes against the hypothesis of trophoblast receptors. The hemochorial placenta in both mice and humans (see Figure 1) supports the hypothesis that micro-traumatic hemorrhages allow for leakage of cells from the placenta to the maternal bloodstream.

## 4. Epitheliochorial Placentas. What Is Known in Cows?

A female cow born with a male twin is known as a freemartin, and this can be explained as the Mc phenomenon. First, in the 1900s, an embryologist from the University of Chicago studied twin fetus development in cows. Lillie injected ink into the umbilical cord of one fetus and demonstrated that blood, as a circuit, goes from the one placenta to the mother’s blood and the second placenta during fetal development. If one of the fetuses is a male and the second is a female, the actions of male hormones result in a female twin with external female genitalia but internal male gonads [48]. At the University of Wisconsin–Madison, Ray David Owen studied a large number of blood samples from pairs of twin calves from all around the USA. In 1945, Owen reported that 90% of freemartins share blood and cells that produce Mc during fetal development [49]. In contrast with Lillie’s explanation, Owen attributed the action of cells rather than hormones to the masculinization of the female cow. It is remarkable that Owen named the phenomenon a mosaic and avoided the term chimerism due to the negative connotations of Greek mythology. In addition, scientific literature spread the terms chimerism and microchimerism to explain how different cell lineages and genomes merge in one organism [50,51].

Feto-fetal Mc during embryo development and its impact on gonadal development in dizygotic twins was first studied in 1962. The study was done in seven Holstein–Friesian calves younger than one day old. The gonad samples for male and female newborns were analyzed using cytological techniques. Interestingly, this data aligns with the hypothesis previously presented by Owen. The group described a 10 to 20% presence of microchimeric cells, XX in males and XY in females, in bone marrow and gonads, particularly in dizygotic twins, suggesting the bidirectional transfer of cells between twins in utero [52]. These animals are known as freemartins, or Taura. This phenomenon has also been reported in goats and, less frequently, in sheep [53,54].

Returning to the topic of fetal–maternal Mc in cattle, it is essential to first understand the morphology and development of the placenta. In the synepitheliochorial type of placenta, typical of ruminants, the multiple layers separate maternal and fetal blood, see Figure 1. Therefore, cffDNA can be less permeable from the placenta to maternal plasma. This scientific paradigm presents contradictory data. First, arguing that the synepitheliochorial placenta is not permeable to cffDNA, by endpoint PCR techniques in eight cows, no cffDNA was found [55]. In contrast, earlier studies have shown varied results in detecting cffDNA during gestation. For instance, in 2006, a report analyzed 32 cows at 30 to 60 days of gestation using nested PCR targeting SRY located in the Y chromosome and achieving 60% accuracy in blood [56]. Another work studied 110 cows from 30 to 242 days of pregnancy using nested PCR targeting SRY, achieving 100% accuracy [57]. Finally, in 2012, a Brazilian group conducted PCR testing on 35 cows at 35 to 48 days, reporting 95% accuracy, though the target gene was not specified [58].

**Figure 2:**
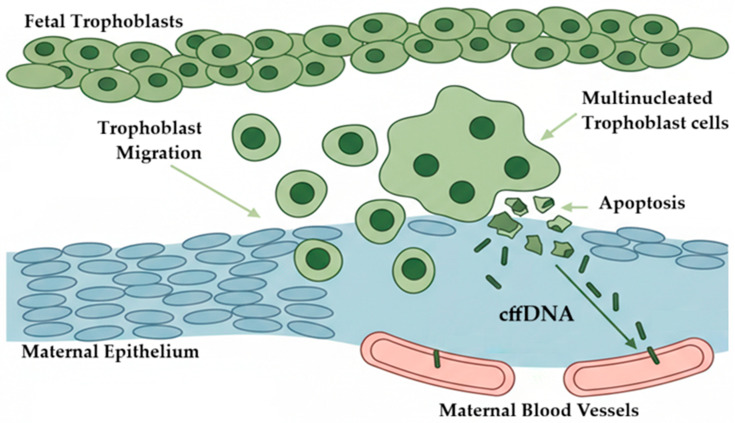
Hypothetical model explaining how fetal cells contribute to Mc in early pregnancy. Trophoblast cells, green color, move to the mother’s uterine surface and merge to form a multinucleated layer, blue color. When these cells die by apoptosis, they release small pieces of cell-free fetal DNA (cffDNA) that reach the mother’s tissue and blood vessels. These multinucleated trophoblast cells are called binucleate cells in the development of the epitheliochorial placenta and syncytial cells in the hemochorial placenta. The arrow indicates the possible flow.

Advancements in digital PCR technologies have demonstrated remarkable sensitivity and precision. For example, De los Santos in 2024 utilized dPCR to detect cffDNA in six out of nine plasma samples from cows between 20 and 61 days post-insemination, with concentrations ranging from 350 to 2300 copies per ml of blood. These findings underscore the potential of dPCR to offer earlier and more accurate insights into fetal genetics. The model presented in this recent research suggests that binucleated cells, after invading the maternal epithelium, undergo apoptosis, releasing cffDNA into the maternal bloodstream as early as the onset of placental invasion, see Figure 2. The novel models suggest that multinucleated cells, formed from trophoblast fusion, act as a source of cffDNA and RNA in maternal blood [59].

In 2011, a Brazilian group showed that free fetal DNA from the fetus can be found in the maternal blood, fragmented; they used PCR techniques and Bos indicus or Zebu as an animal model [60]. Another research group from Germany used blood samples from dairy cattle or Holstein–Friesians, and sequenced DNA fragments coming from the fetus or placenta isolated from the maternal blood by DNA sequencing techniques [61]. These two articles demonstrated that cffDNA can be found in cattle during pregnancy despite the difference in the placenta structure of ruminants and other mammals, see Figure 1. The accuracy of PCR techniques is lower than in humans, and some researchers argue that the synepitheliochorial placenta prevents cffDNA from being found in cows’ blood during gestation [55]. In addition, real-time PCR techniques show a 98.5% accuracy in the fetal sex determination in humans [62]. In contrast, the end-point PCR reflects an accuracy of only 63.6% [58] in cattle, and 87.5% in another bovine study [63]. The biological fate and rationale of this phenomenon remain an ongoing topic of exploration in animal science research. A new hypothesis points out that cffDNA in maternal blood originates from the apoptotic process of binuclear trophoblast cells [59].

Additional information came from the study of proteins; the multinucleated cells (trinucleate in cow and deer, syncytial in sheep and goats) can secrete specific proteins by exocytosis from the fetal-formed, binucleate cell-delivered granules into the maternal organism. The placental lactogen (PL) is present in maternal serum in concentrations around 1–2 ng/mL and in fetal serum at 5–12 ng/mL [64]. This data is consistent with the hypothesis that binucleate placenta cell migration accomplishes the delivery of bovine PL to the maternal blood circulation [64]. The mRNA encoding the bovine PL gene was first detected in the placenta around day 20 of gestation, correlating with the first appearance of binucleate cells [65].

In goats, also with a cotyledonary placenta, Mc has not been extensively studied [66]. Both species share similar placental structures, including binucleate trophoblast cell migration into maternal tissue [64], highlighting a clear gap in the literature.

## 5. What Has Been Proven in Pigs and Horses?

Pigs, featured in five studies, are primarily examined in the context of xenotransplantation. Since pigs are considered potential organ donors for humans, understanding how pig cells behave in maternal systems is crucial for assessing safety and regulatory concerns.

The placenta in pigs is epitheliochorial, in which the chorion of the fetal placenta is in contact with the epithelium of the uterus, see Figure 1. Two studies show the natural Mc in pigs. In the first, following the injection of human cord blood-derived cells in swine fetuses between 40 and 43 days of gestation, the presence of these cells was detected after delivery in 78% of unmanipulated littermates. Moreover, injected human cells were detected in various pig tissues eight months after birth, including the kidney (56%), spleen (33%), thymus (11%), and heart (22%) [67]. The second study argues that there is no evidence for microchimerism in porcine fetuses and their mothers. This study did not find Mc signaling after an inspection of both blood and solid organs. The authors support that the epitheliochorial structure of the porcine placenta somehow prevents cellular exchange during gestation [68].

Horse: Only one article refers to cffDNA in mare plasma [69]. No data for feto-to-feto Mc in the literature was found at the time to publish this article. This opens the discussion about whether the epitheliochorial and diffuse types of placenta prevent Mc or if the apparent absence is due to a lack of scientific interest. In any case, a second gap in literature was found.

## 6. Discussion

The publications show that natural feto-maternal Mc is not limited to humans and mice. Instead, Mc appears to be widespread among placental mammals and takes place with no human intervention, also known as natural Mc [2]. A single article about dogs with natural feto-maternal Mc was found [70]. These findings support the idea that chimerism is not merely a peculiarity observed in laboratory models, but rather a fundamental biological process across mammalian species. More data is needed to confirm this trend, which suggests that feto-maternal MC does not appear to depend strictly on placental type, as illustrated in Figure 1.

Taking all the information into account, a new biological model is presented in Figure 2. This hypothesis proposes that, during early placentation and at the cellular level, invasive trophoblasts interacting with epithelial cells give rise to binuclear cells (syncytiotrophoblasts) in hemochorial placentas or to binuclear/trinuclear giant cells in epitheliochorial placentas. These unique cells, after extraembryonic development, undergo cell death and release cffDNA, which in most articles is referred to as Mc. This hypothesis explains why the literature has not reported that Mc occurs before attachment or placenta implantation. This hypothesis could be tested by assessing placental proteomic markers in the uterus at early placentation in parallel with circulating cffDNA markers.

## 7. Conclusions

As I account here, trophoblasts, multinucleated cells, erythrocytes, lymphocytes, DNA, RNA, and proteins can pass across the placenta to the maternal blood in mammals. These observations were documented using a variety of molecular technologies, including PCR for detecting Y-chromosome fragments of DNA, FISH for tissue detection, and the analysis of STR and HLA alleles to track genetic variants across generations, as well as cell filtration. These observations are obvious across mammals in varied species. Mc phenomena seem to be independent of placental structure. Despite these molecular techniques, there is a literature gap for capra and equus, which represent epitheliochorial cotyledonary and diffuse placentas, respectively. endotheliochorial placentas, typical of canidae and felidae, also show a lack of studies on Mc.

The literature presents several hypotheses regarding the origin of Mc in both hemochorial and epitheliochorial placentation. A cellular model is proposed in this article and remains to be tested in vivo across different species. This may help unify the Mc phenomenon in mammals if it proves to be a universal process, with potential impact on diverse fields such as immunology and embryology. This can open new innovative research lines, for instance, gene transfer between embryos during gestation or from the fetus to the mother during pregnancy.

## Data Availability

No new data were created or analyzed in this study.

## Abbreviations

The following abbreviations are used in this manuscript:

CD34	Cluster Differentiation 34
cffDNA	Cell-free Fetal DNA
Mc	Microchimeric
FISH	Fluorescent In Situ Hybridization
HLA	Human Leukocyte Antigen
Mc	Microchimerism
PCR	Polymerase Chain Reaction
PL	Placental Lactogen
STR	Sort Tandem Repeat
TfR	Transferrin Receptors
